# Digital Therapeutic FEMANEA® for Women with Stress and Mixed Urinary Incontinence: A Randomized Controlled Pilot Study

**DOI:** 10.1007/s00192-026-06670-z

**Published:** 2026-04-30

**Authors:** Anna Schneiderbanger, Katharina Arndt, Hannah Arnold, Matthias W. Beckmann, Vesna Bjelic-Radisic, Hendrik Borgmann, Stefanie Burghaus, Emilia Fieseler, Thomas Fink, Michael Gedamke, Matthias Goetze, Anna-Lena Nachtigal, Maja Vorwerk, Bernd Wullich, Verena Lieb

**Affiliations:** 1 Department of Urology and Pediatric Urology, Uniklinikum Erlangen, Friedrich-Alexander-Universität Erlangen-Nürnberg, Erlangen, Germany; 2 Continence and Pelvic Floor Center, Uniklinikum Erlangen, Friedrich-Alexander-Universität Erlangen-Nürnberg, Erlangen, Germany; 3 Department of Urology and Pediatric Urology, Faculty of Health Sciences, Brandenburg Medical School Theodor Fontane, Brandenburg an der Havel, Germany; 4 Urology Senden, Ulm, Germany; 5 Department of Gynecology and Obstetrics, Uniklinikum Erlangen, Friedrich-Alexander-Universität Erlangen-Nürnberg, Erlangen, Germany; 6 Breast Unit, Dept Senology, Helios University Hospital, Wuppertal, University Witten/Herdecke, Wuppertal, Germany; 7 Department of Gynecology and Urogynecology, Sana Hospital Lichtenberg, Berlin, Germany; 8 FARCO-PHARMA GmbH, Cologne, Germany

**Keywords:** Digital therapeutics, Female urinary incontinence, ICIQ-UI SF, Patient-reported outcomes, Pelvic floor therapy, Women’s health

## Abstract

**Introduction and Hypothesis:**

This exploratory pilot study provided initial evidence of the effectiveness of the digital therapeutic (DTx) FEMANEA® for the conservative treatment of stress urinary incontinence (SUI) and mixed urinary incontinence (MUI) with a predominant stress component in women and is intended to prepare a further, more in-depth subsequent randomized controlled trial (RCT).

**Methods:**

Between May and October 2024, 81 patients (women at least 18 years old; SUI or MUI) were enrolled at five centers and randomized into an intervention group (IG, *n* = 34) or a control group (CG, *n* = 40). Both groups received standard conservative treatments (i.e., reality of care), the IG additionally had immediate access to the DTx FEMANEA®. Outcome parameters were assessed at baseline, mid-treatment (45 days), and post-treatment (90 days) using the International Consultation on Incontinence Questionnaire-Urinary Incontinence Short Form (ICIQ-UI SF), King’s Health Questionnaire (KHQ), bladder diary, 24-h pad test, Patient Activation Measure (PAM-13), and app usage metrics. Data collection was carried out electronically with eCRFs (electronic case report forms) and ePROs (electronic patient reported outcomes).

**Results:**

The final analysis included 74 patients (dropout rate 6.2%). At baseline, symptom burden was slightly higher in the IG than in the CG (ICIQ-UI SF: T0 IG 10.7 ± 4.3, CG 9.2 ± 3.0; *p* = 0.09). The CG showed no significant improvement in symptoms (T2 8.3 ± 2.7; Δ = –0.9, *p* = 0.051), whereas the IG demonstrated a significant and clinically relevant reduction in symptoms (T2 8.2 ± 4.7; Δ = –2.2; *p* = 0.001), exceeding the minimal important difference (MID) of 1.46 points. Additionally, the KHQ subscale 2 “incontinence impact on life” observed a significant improvement (IG –19.8 ± 18.7 vs. CG –5.3 ± 19.8; *p* = 0.002), with IG improvements also being clinically relevant and surpassing the MID by a factor of four.

Daily incontinence episodes decreased by 79.7% in the IG versus 48.3% in the CG (each group *p* < 0.01). Urinary loss volume revealed a 44.0% reduction in the IG, whereas CG values slightly increased. The mean app use in the IG was 2.6 ± 1.9 days per week per patient.

**Conclusions:**

Use of the DTx FEMANEA® resulted in clinically and statistically significant improvements in UI symptom burden (ICIQ-UI SF) and quality of life (KHQ subscale 2). These initial clinical data are intended to provide preliminary indications and prepare for the subsequent trial to be conducted.

**Supplementary Information:**

The online version contains supplementary material available at https://doi.org/10.1007/s00192-026-06670-z.

## Introduction

In general, urinary incontinence (UI) is defined as any involuntary loss of urine and is one of the most common clinical conditions [1–3]. Involuntary loss of urine can significantly restrict the everyday activities of sufferers and impair their quality of life [4–7]. In some cases, this leads to deterioration or severe impairment of quality of life and is associated with socioeconomic consequences [2].

In Germany the prevalence of UI in women older than 18 years is 48.3%, with women being affected more often than men [8]. From a pathophysiological point of view, female UI can be categorized into different types. In Germany, stress UI (SUI) is the most common form of UI in women aged 18 and over, with a prevalence rate of 24.5% [8]. However, the prevalence of SUI varies depending on the age group, increases with age, and peaks between the ages of 40 and 59 years, correlating with the onset of menopause [8]. SUI is the involuntary loss of urine during physical exertion without a simultaneous urge to urinate [1]. It can be triggered by any mechanical stress that leads to an increase in pressure in the abdominal cavity. This includes, for example, physical activity, exertion, coughing, or sneezing. Another form of incontinence is urge UI (UUI), which is characterized by involuntary loss of urine accompanied by an imperative urge to urinate, and mixed UI (MUI), which includes symptoms of both SUI and UUI [1]. The prevalence of MUI (13.1%) and UUI (10.7%) in Germany is significantly lower than that of SUI [8].

After a differentiated diagnosis of UI, conservative nonsurgical therapy methods are the first-line approach in clinical practice and are also recommended in guidelines [6, 7], as they usually carry the lowest risk of harm. These include clinical measures such as the treatment of comorbidities, the adjustment of medication, the treatment of constipation and the use of medical aids. Lifestyle-related interventions, such as reducing body weight or caffeine consumption, are also part of the conservative therapeutic approach [3, 6, 7]. In particular, patients with SUI or MUI should be offered pelvic floor muscle training (PFMT) for at least 3 months as the gold standard. Drug therapy in the form of local treatment with estrogens may also be considered. Surgical therapy should be used only if conservative therapy does not achieve the desired effect [3, 6, 7]. However, there is a gap between the standard of care (SOC) (as indicated in the German guideline “Urinary incontinence in women”) and the reality of care in Germany.

Digital solutions for patients with UI are currently being scientifically investigated. Undergoing PFMT independently via a Swedish mobile app could improve or simplify access to health care [9]. The digital therapeutic (DTx) FEMANEA® was developed to provide medical treatment for patients with diagnosed UI. FEMANEA® is an exercise DTx that is available daily over 90 days and is specifically adapted to the individual needs of patients.

A two-stage procedure was selected in accordance with the Fast-Track Process for Digital Health Applications (DiGA) of the German Federal Institute for Drugs and Medical Devices (BfArM) to demonstrate the positive care effect of the DTx FEMANEA® [10]. The aim of this exploratory, randomized, controlled, multicenter pilot study is to generate initial clinical data following CE-marking of DTx FEMANEA® and to obtain initial evidence of its potential benefits for patients with UI. The DTx FEMANEA® is currently not commercially available. This approach is intended to prepare a further, more in-depth subsequent randomized controlled trial (RCT) with a view to obtaining permanent approval as a DiGA in Germany. The medical intention of FEMANEA® is to close the addressed care gap in female UI.

## Materials and Methods

### DTx Application FEMANEA®

FEMANEA®, a DTx, was launched in April 2024. It is classified as a class I medical product according to the Medical Device Regulation (MDR) and meets all requirements of the Digital Healthcare Act (DVG). FEMANEA® provides a daily digital PFMT program for 90 days for women diagnosed with UI.

**Fig. 1:**
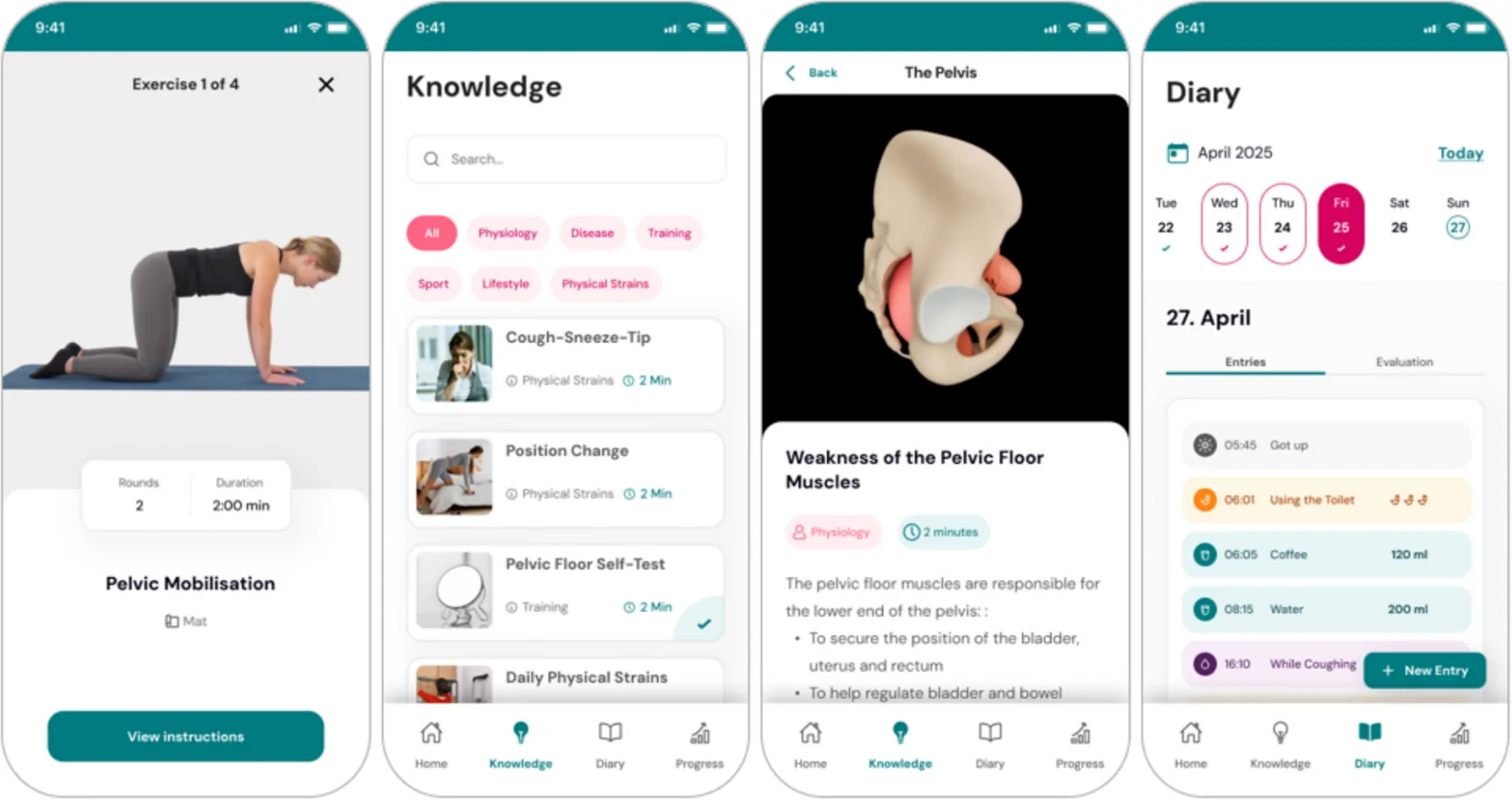
Insights into FEMANEA®. An example of an exercise (a therapy unit consists of several exercises); an overview of the knowledge area and the micturition diary are shown

The DTx FEMANEA® is aimed specifically at women with SUI and MUI with a predominant stress component. Therefore, most therapy consists of targeted physiotherapeutic interventions, especially PFMT. Within an algorithm-controlled system, approximately 246 different exercises were presented to the patients. In addition, behavioral therapy components (e.g., a bladder diary for self-monitoring, relaxation techniques) were integrated into the treatment plan. Educational content in the form of text and video provides the necessary knowledge about the condition and anatomy supporting the treatment of the underlying indication (Fig. 1). The PFMT plan is continuously adapted to the individual needs of the user over the course of the therapy based on personal information on performance and perceived exertion. At present, FEMANEA® is available in Germany and in the German language, with potential for future internationalization.

### Patients and Study Design

An exploratory, prospective, multicenter, stratified (by study center; randomized 1:1), single-blind, controlled pilot study was conducted. Female patients (at least 18 years old) with SUI or with MUI with a predominant stress component were included (ICD-10 codes N39.3, N39.47, N39.48; Ingelman–Sundberg severity grade 1 or 2). Prior ultrasound assessment with postvoid residual measurement and vaginal positioning had to have been conducted by board-certified urologists or gynecologists. Additionally, patients were required to have the cognitive ability to understand the nature and implications of data analysis and the training program and fulfill the technical requirements (including internet access and a compatible playback device) (Supplemental Table 2).

Randomization was performed using a centralized electronic data capture system (EDC system) via stratified block randomization by center, based on a pre-established list by the statistician. Stratification ensured a balanced allocation of patients between the intervention group (IG) and control group (CG) across centers. Randomization was initiated by the investigator after obtaining written informed consent, confirming eligibility and conducting the baseline-assessment (screening-visit and medical history), with immediate allocation feedback.

Both groups were offered reality of care (i.e., the physicians did not alter their usual practice). Conservative treatment methods include clinical measures (e.g., treatment of constipation, use of medical aids), lifestyle interventions (e.g., reduction in caffeine intake, weight loss), individualized behavioral and physiotherapy, and local estrogen therapy [3, 6, 7].

The IG additionally received immediate access to the DTx FEMANEA® by being provided with an access code for the DTx after diagnosis and randomization. It is only possible to use the training program with the registration code. The CG received reality of care only. After the 90-day treatment period, the CG received access code to the DTx FEMANEA®.

Allocation concealment was ensured until the point of randomization as the final step of the first study visit. Therefore study personnel were blinded to treatment allocation up to the moment of randomization; no blinding was maintained thereafter. The statistician remained blinded to group allocation during the analysis after database lock.

All patients provided written informed consent prior to randomization, including a data protection consent declaration and were offered financial compensation for participating in the study and completing the questionnaires in the form of a 100 Euro gift voucher.

### Endpoints and Assessment

As mentioned before, a two-stage procedure was selected in accordance with the Fast-Track Process for DiGA of the BfArM to demonstrate the positive care effect of the DTx FEMANEA®. The first stage comprised a pilot exploratory randomized controlled trial designed to provide initial insights into the potential medical benefit of the DTx FEMANEA® and to inform the design of the subsequent full-scale RCT (second stage), particularly regarding sample size estimation, measurement instruments, primary endpoints and recruitment strategies [10].

The endpoints included validated patient-reported outcome measures (PROMs) used for UI symptom burden (International Consultation on Incontinence Questionnaire-Urinary Incontinence Short Form, ICIQ-UI SF) and health-related quality of life (incontinence impact on life) (King’s Health Questionnaire Subscale 2, KHQ S2) [11–14].

Further endpoints included the frequency of involuntary urinary loss over three consecutive days quantified using a bladder diary, the amount of involuntary urinary loss assessed by a 24-h pad test and active patient activation measured by the Patient Activation Measure-13 (PAM-13) [6, 7, 15]. Finally, app usage was analyzed.

Data were obtained at T0 = 1 + 7 days (baseline), T1 = 45 ± 5 days, and T2 = 95 ± 5 days via electronic case report forms (eCRFs) and electronic patient reported outcomes (ePROs).

### Statistical Analysis

The statistical analysis included an intragroup comparison using a *t*-test to determine whether the differences between the values at T0 and T2 were significant. Intergroup comparisons were carried out via *t*-tests for larger samples or Mann–Whitney *U* tests or permutation tests for smaller samples. Effect size (Cohen’s *d* or rank-biserial correlation) was also calculated to assess the practical relevance of the results. When appropriate, ANCOVA (analysis of covariance) and mixed-model analyses were performed. The primary analyses were conducted in the intention-to-treat (ITT) population, defined as all randomized patients; patients with missing baseline values (T0) were excluded. Missing data were handled using last observation carried forward (LOCF) and jump-to-reference predictive mean matching (J2R-PMM). For J2R-PMM, missing values in the IG were imputed from the CG distribution at the same time point. Clinical relevance was assessed via predefined thresholds (minimal clinically important difference (MCID) or minimal important difference (MID)). The significance level was set at *α* = 0.05 for all analyses and tests. The analyses and evaluations were implemented using Python (version 3.11).

## Results

The following results are based on the complete-case analysis. The imputed datasets were used in supplementary sensitivity analyses only.

### Patient Characteristics

**Fig. 2:**
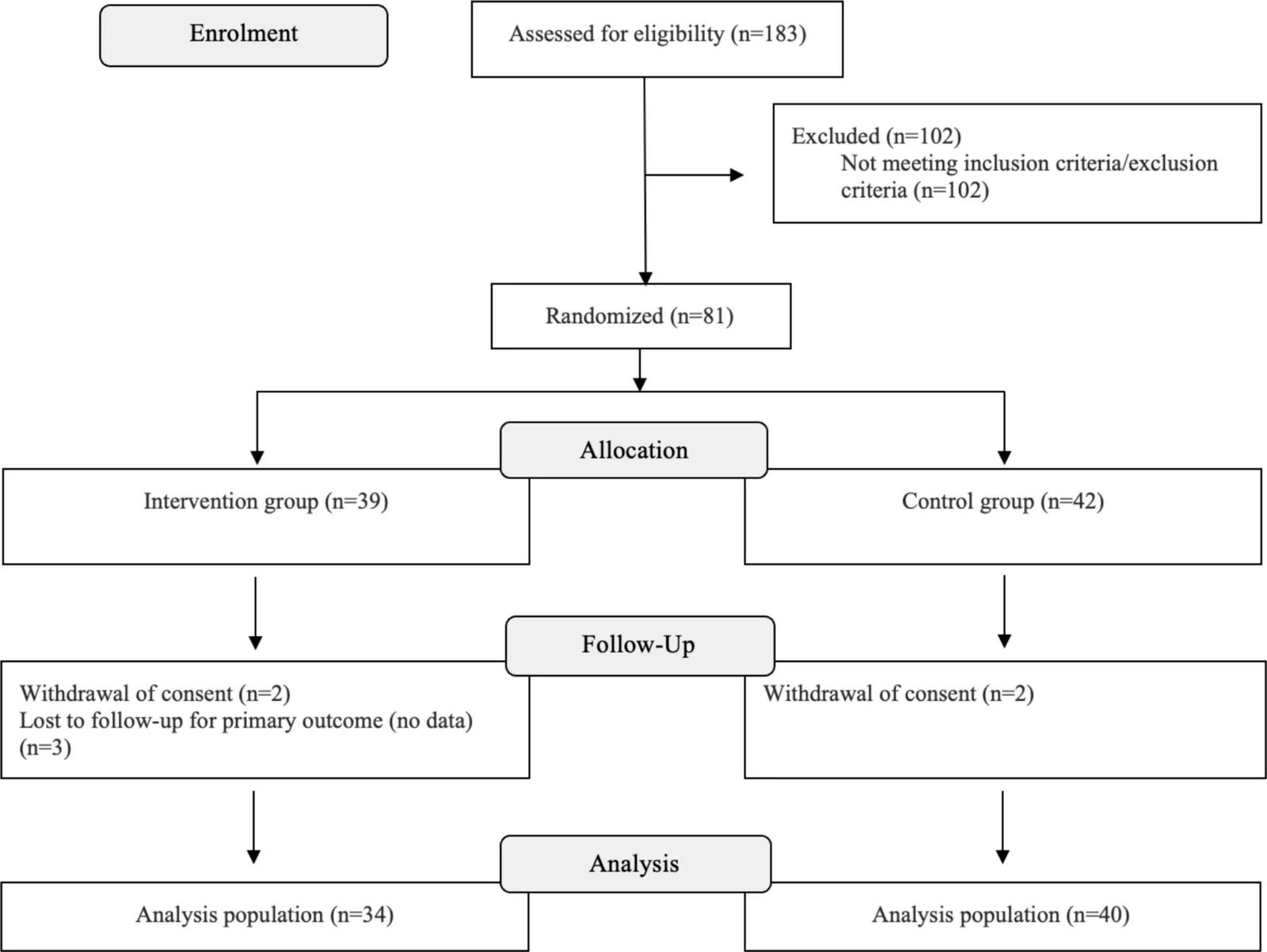
Consolidated Standards of Reporting Trials (CONSORT) flow diagram

Between May and October 2024, a total of 183 female patients were approached at seven study centers. After screening for inclusion and exclusion criteria and obtaining informed consent, 81 patients were enrolled from five study centers. The dropout rate was 6.2% (reason: withdrawal of consent), which was evenly distributed between both groups, resulting in an analysis population of 74 patients: 34 patients were randomized to the IG, and 40 were randomized to the CG (Fig. 2).

Baseline patient characteristics were equally distributed between the groups (Table 1 and Supplemental Tables 3, 4, 5, and 6).

**Table 1:** Patient baseline characteristics

	All patients *n* = 74	CG *n* = 40	IG *n* = 34	*p* value* CG vs. IG
**Age distribution [n (%)]**				
18–65 years	70 (94%)	38 (95%)	32 (94%)	1
> 65 years	4 (6%)	2 (5%)	2 (6%)	-
**Age [years; M (SD)]**				
All patients	48.4 (10.3)	47.9 (9.8)	48.9 (10.9)	0.671
18–65 years	47.0 (8.8)	46.7 (8.4)	47.4 (9.3)	0.727
> 65 years	72.0 (4.2)	71.0 (4.2)	73.0 (5.7)	-
**BMI (kg m**^**−2**^**; M (SD)]**	25.4 (5.2)	25.3 (5.0)	25.4 (5.6)	0.960
**Indication [n (%)]**				
N39.3 stress urinary incontinence	74 (100%)	40 (100%)	34 (100%)	
N39.47 recurrent urinary incontinence	-	-	-	
N39.48 other specified urinary incontinence	-	-	-	
**Incontinence type [n (%)]**				0.814
SUI	50 (68%)	28 (70%)	22 (65%)	
MUI	24 (32%)	12 (30%)	12 (35%)	
**Ingelman-Sundberg severity grade [n (%)]**				0.504
Severity grad I	56 (76%)	32 (80%)	24 (71%)	
Severity grade II	18 (24%)	8 (20%)	10 (29%)	
**Duration of UI [months; M (SD)]**	90.7 (78.8)	84.4 (67.7)	98.12 (90.7)	0.471
**Number of births per woman [M (SD)]**	1.9 (1.0)	2.0 (1.0)	1.7 (0.9)	0.225
**Births in the last 12 months [n (%)]**				
Yes	4 (5%)	3 (8%)	1 (3%)	
No	70 (95%)	37 (92%)	33 (97%)	
**Menopause**				0.55
Yes	31 (42%)	15 (37.5%)	16 (47.1%)	
No	43 (58%)	25 (62.5%)	18 (52.9%)	
**Graduation**				0.836
None	1 (1%)	-	1 (3%)	
Basic secondary school diploma	3 (4%)	2 (5%)	1 (3%)	
Secondary school diploma	24 (32%)	12 (30%)	12 (35%)	
Universities of applied sciences entrance qualification	11 (15%)	6 (15%)	5 (15%)	
General university entrance qualification	33 (45%)	19 (17.5%)	14 (41%)	
Other	2 (3%)	1 (2.5%)	1 (3%)	
**Vocational training**				0.487
None	1 (1%)	-	1 (3%)	
Training	39 (53%)	20 (50%)	19 (56%)	
Bachelor’s degree	6 (8%)	2 (5%)	4 (12%)	
Master’s degree/Diploma	19 (26%)	13 (33%)	6 (18%)	
Doctor’s degree	4 (5%)	2 (5%)	2 (6%)	
Other	5 (7%)	3 (8%)	2 (6%)	
**Educational status**				0.573
Employed	57 (77%)	29 (73%)	28 (82%)	
Civil servant	5 (7%)	3 (8%)	2 (6%)	
Self-employed	2 (3%)	2 5%)	-	
Housewife	5 (7%)	3 (8%)	2 (6%)	
Pensioner	5 (7%)	3 (8%)	2 (6%)	
**Marital status**				0.400
Married	50 (68%)	29 (73%)	21 (62%)	
Married (separated)	1 (1%)	-	1 (3%)	
Single	11 (15%)	6 (15%)	5 (15%)	
Divorced	11 (15%)	5 (13%)	6 (18%)	
Widowed	1 (1%)	-	1 (3%)	
**Active in sports**				1
Yes	53 (72%)	29 (72.5%)	24 (70.6%)	
No	21 (28%)	11 (27.5%)	10 (29.4%)	

**Abbreviations**: *CG* control group, *IG* intervention group, *UI* urinary incontinence, *SUI* stress UI, *MUI* mixed UI, *n* number, *M* mean value, *SD* standard deviation, *BMI* body mass index *calculated using the *T* test for metric variables and the chi-square test or Fischer test for nominal variables; “-” = no information was available if the number of cases was too small

In the present study, 70.3% of all patients were prescribed physiotherapy at T0, with similar proportions in the CG (72.5%) and IG (67.6%). However, a greater percentage of patients in the CG than in the IG underwent physiotherapy at T2 (CG 47.5% vs. IG 29.4%), with an overall rate of 39.2% (Supplemental Table 7).

### ICIQ-UI SF

**Fig. 3:**
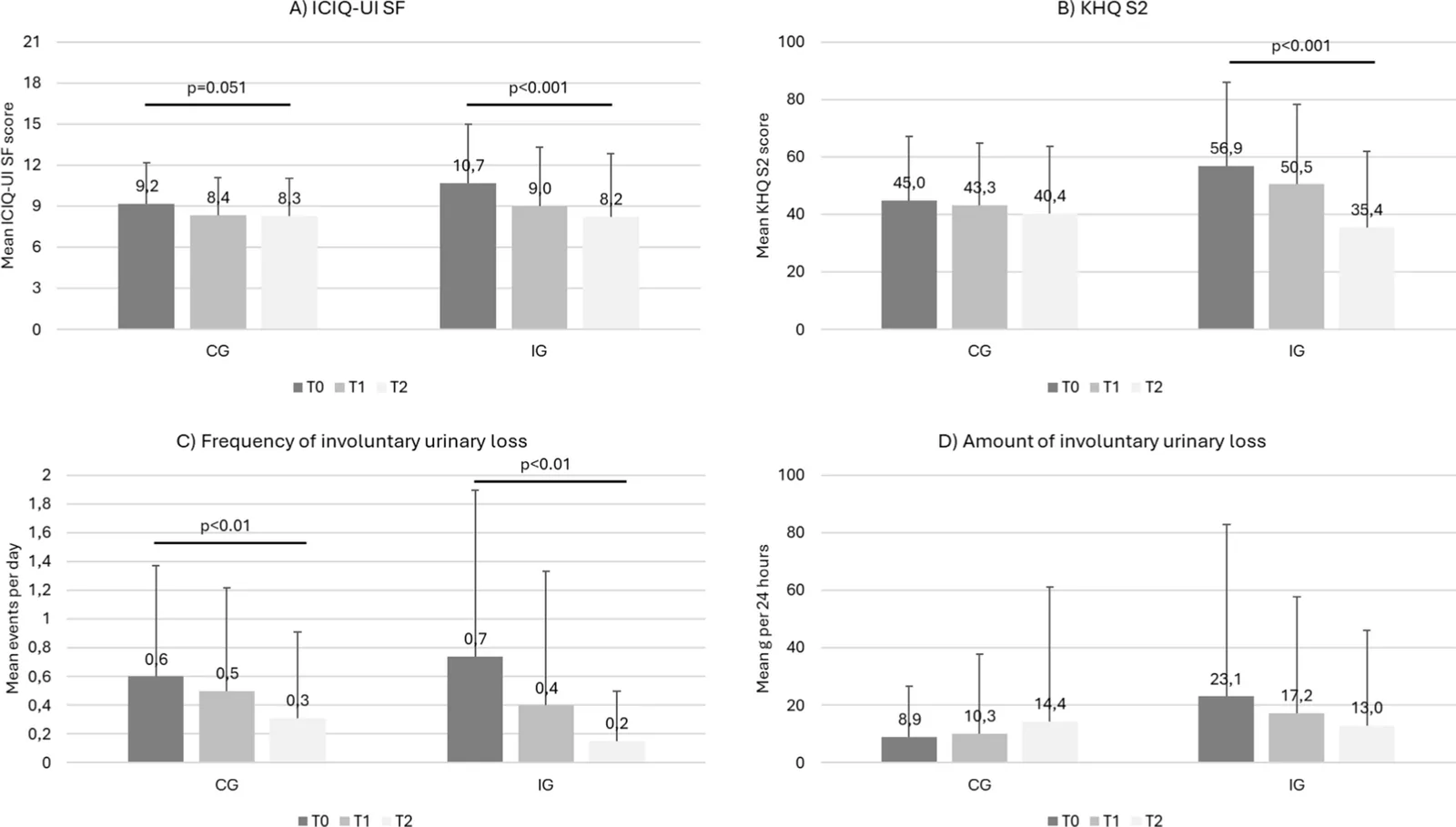
Main findings of the study

The ICIQ-UI SF was used to assess the UI symptom burden. At baseline, the IG tended to have more severe symptoms than the CG did (IG 10.7 ± 4.3, CG 9.2 ± 3.0, *p* = 0.091). Patients in the CG showed almost no significant improvement from baseline (T2 8.3 ± 2.7; Δ(T0, T2) 9.6%, *p* = 0.051), whereas patients in the IG demonstrated continuous and significant improvement throughout the study period (T2 8.2 ± 4.7; Δ(T0, T2) 23.2%, *p* < 0.001). The reduction in the ICIQ-UI SF score was significantly greater in the IG than in the CG (IG −2.2 ± 2.8; CG −0.8 ± 2.6;* p* = 0.037) (Fig. 3 and Supplemental Table 8). In the complete-case mixed-model analysis, the baseline-adjusted between-group difference at T2 was −1.41 points (95% CI −2.65 to −0.18; *p* = 0.025), indicating significantly greater inprovement in the IG than in the CG. This effect showed a mean effect size of −0.51 (Cohen’s *d*) (Supplemental Table 10). The observed improvement in the IG was 1.5 times greater than the MID of 1.46 points [16], indicating a clinically relevant improvement regarding the UI symptom burden.

### KHQ

The KHQ was used to measure the health-related quality of life and the subscale 2 “incontinence impact on life” (KHQ S2) is individually validated and evaluable as a subscale [13]. At baseline, the IG had a higher score on the KHQ S2 “incontinence impact on life” than did the CG (IG 56.9 ± 29.1; CG 45.0 ± 22.1; *p* = 0.056). While the CG showed no significant improvement (T2 40.4 ± 23.5; Δ(T0, T2) 10.3%; *p* = 0.110), the IG demonstrated continuous and significant improvement throughout the study period (T2 35.4 ± 26.7; Δ(T0, T2) 37.7%; *p* < 0.001). The decrease in KHQ S2 was significantly greater in the IG than in the CG (IG −19.8 ± 18.7; CG −5.3 ± 19.8; *p* = 0.002) (Fig. 3 and Supplemental Table 8). In the complete-case mixed-model analysis, the baseline-adjusted between-group difference at T2 was −14.92 points (95%−CI −23.89 to −5.96; *p* = 0.001), indicating significantly greater improvment in the IG compared to the CG (Cohen’s *d* = −0.76) (Supplemental Table 11). The improvement in the IG exceeded the MID threshold of 5 points [17, 18] by a factor of almost 4, indicating a clinically relevant app-induced improvement.

### Frequency and Amount of Involuntary Urinary Loss

To obtain information on the frequency of involuntary urinary loss, a bladder diary was kept for 3 days. At baseline, the IG experienced a slightly greater frequency of events per day than did the CG (IG 0.7 ± 1.2; CG 0.6 ± 0.8, *p* = 0.567). Over the course of the study, both groups presented a significant reduction in the number of events per day (T2 IG 0.2 ± 0.4; CG 0.3 ± 0.6; both *p* < 0.01), with a 48.3% reduction in the CG compared to 79.7% reduction in the IG (Fig. 3 and Supplemental Table 9).

The amount of involuntary urinary loss measured by a 24-h pad test at baseline was approximately 2.5 times greater in the IG than in the CG (IG 23.1 ± 59.8; CG 8.9 ± 17.9, *p* = 0.189). While the amount of involuntary urinary loss increased over the study period in the CG (T2 14.4 ± 46.8, *p* = 0.303), the IG showed an average reduction of approximately 44.0% (T2 13.0 ± 33.1, *p* = 0.161) (Fig. 3 and Supplemental Table 9). In the complete-case mixed-model analysis, the baseline-adjusted between-group difference at T2 was −14.22 units (95%−CI −29.05 to 0.60; *p* = 0.060), with a moderate effect size (Cohen’s *d* =  −0.43), indicating no statistically significant difference between groups at T2, but a clear trend in favor of the IG (Supplemental Table 12).

### PAM-13

Patient participation measured by the PAM-13 was rated as very high throughout the study. The baseline mean was 95.2 ± 2.7% across all participants. No significant changes were observed in either group over the entire duration of the study (IG *p* = 0.810, CG *p* = 0.135).

### App Usage Data

During the entire study period, the DTx FEMANEA® was used by all patients in the IG for a total of 1142 days. On average, patients used the DTx for 33.6 out of 90 days (mean 33.6, SD 24.6; median 27, interquartile range (IQR) 42), corresponding to a weekly usage frequency of 2.6 ± 1.9 days per week per patient. This finding is consistent with the self-reported usage time of 2.8 ± 2.0 days per week per patient. In the IG, the frequency of therapy within 12 weeks was therefore approximately six times greater than that in the CG, assuming two physiotherapy sets (2 recipes with 6 units) and considering that only 47.5% of patients in the CG actually underwent physiotherapy (see the “Patient characteristics” section and Supplemental Table 7).

## Discussion

This exploratory pilot study provides initial indications of the potential of the DTx FEMANEA® for patients with SUI or MUI with a leading stress component. The findings will be incorporated into the planned RCT and will be used to select the final primary and secondary endpoints, to define proper sample size estimation, measurement instruments, and recruitment strategies. This procedure is part of the two-stage procedure of the BfArM in Germany. The final intention is permanent inclusion in the DiGA directory of the BfArM.

The results revealed a statistically significant and clinically relevant improvement in health-related quality of life (incontinence impact on life, KHQ S2) and in UI symptom burden (ICIQ-UI SF) after 90 days of using the DTx FEMANEA® in direct comparison to reality of care in Germany. Between-group comparisons consistently showed significantly greater improvements in the IG for both endpoints (KHQ S2, ICIQ-UI SF) in mixed-model analyses. In addition, the frequency of involuntary urinary loss in the IG was significantly reduced during the study period. This trend was also observed in the amount of involuntary urinary loss in the IG, whereas opposite trends were observed in the CG. Effect size estimates further supported the magnitude and clinical relevance of these differences between the groups. In summary, these findings indicate the potential benefit of DTx-based therapy components. This is in line with the results already described in other studies, which indicate that unsupervised, home-based or internet-based self-exercise programs such as PFMT with mobile applications are a promising method to complement or replace physiotherapy treatment. This approach has the potential to improve patient care or symptoms, patient satisfaction and patient adherence, or at least achieve results equivalent to those of conventional treatment [9, 19–23].

The slightly different number of participants in the two groups (IG 34, CG 40) resulted from the randomization applied, which was stratified by study center. The average age, 48.9 ± 10.9 years in the IG and 47.9 ± 9.8 years in the CG, corresponds to the average age in Germany at which UI reaches its peak [8]. In both groups, severity grade I according to Ingelman-Sundberg (*n* = 56, 76%) was overrepresented compared with severity grade II (*n* = 18, 24%) but was equally distributed between the two groups (IG and CG). With respect to the type of incontinence, SUI accounted for the largest proportion, with a total of 68% of the included patients having SUI. This corresponds approximately with the distribution of incontinence types in the population, and SUI is the most common type of incontinence in women [8].

Despite these promising findings, there are several potential limitations that must be considered. First, both the CG and the IG received reality of care, and the IG additionally received the DTx FEMANEA®. Owing to a potentially varying “reality of care” depending on the practitioner the CG represents a heterogeneous and non-standardized benchmark, which can lead to variations in treatment intensity and limit both the internal validity and the reproducibility of the results. A survey conducted in 2024 among doctors in Germany (presented at the 35th Congress of the German Continence Society, November 2024; data not shown) revealed that more than 50% of doctors prescribe physiotherapy only for every second patient and that there are significant waiting times for physiotherapy treatment. Since we also collected data on waiting times for physiotherapy as part of the pilot study, we were able to confirm that waiting times were comparable in both groups, which indicates that overall homogeneity of reality of care in both groups is given and that we have tested FEMANEA® against real-world conditions. Second, the statistician conducting the analysis was blinded, but the assisting staff was not. The lack of blinding of the assisting staff was protected against systematic bias by the study flow, the digital completion of the questionnaires by the patients outside the centers, the properties of the EDC system and the stratified randomization by study centers. In the same way, the internal processes of equal treatment of both groups, the storage of the randomization lists in the digital system and the process of exporting the data from the EDC system with closing of the database before the prespecified analysis protected against systematic bias. Third, stratification by study center excluded bias induced by specialized treatment modalities or levels of care. Adherence to the reality of care of the investigators was an appropriate control group, as no further influence was exerted. Nevertheless, adherence to the DTx FEMANEA® varied among participants (average use 2.6 ± 1.91 days per week), which may have influenced the observed treatment effects. The close monitoring and layman-friendly language of the concomitant diseases and medications at all data analysis points made it possible to exclude confounding variables such as potentially influencing medication at the endpoints with certainty. We could exclude an effect on the outcome variables due to a new start of causal concomitant therapy with equal distribution in both groups by a separate analysis. However, this also explains the improvement over the study period in the CG. Regardless, with relatively small sample sizes, the improvement in health-related quality of life was significantly greater in the group without physiotherapy in the IG than in the CG, and the improvement in the IG was above the MID [17, 18]. Exclusively self-reported data can lead to an overestimation of actual symptom improvement due to confirmation bias. However, additional verification of symptom improvement was achieved by the 24-h pad test and the bladder diary to increase the reliability of the results (Fig. 3 and Supplemental Table 9). The selection and use of several variables that showed a consistent picture in the context of the expected changes and fluctuations confirm the usefulness of the DTx FEMANEA®. However, given the exploratory nature of the pilot study, the limited sample size, and the statistical models used, these results should be interpreted as preliminary and hypothesis-generating. They suggest a potential benefit of FEMANEA® as a DTx, as well as a possible contribution to closing the gap in care for conservative treatment of SUI.

The data from this pilot study serve as initial evidence of the potential of the DTx FEMANEA® to provide medical benefits as a DTx in the area of conservatively managed UI in women. Notwithstanding the aforementioned limitations—or perhaps precisely because of them—the pilot study yields valuable insights into DTx-based interventions and establishes a robust scientific foundation for the design and implementation of the forthcoming randomized controlled trial.

## Conclusion

The results of this exploratory pilot study indicate a statistically significant and clinically relevant improvement in health-related quality of life (incontinence impact on life) and in the UI symptom burden after 90 days of access to the DTx FEMANEA®. These findings provide initial indications of the potential of the DTx FEMANEA® for patients with SUI and MUI with a leading stress component and suggest a possible contribution to addressing the health service gap in the conservative treatment of SUI. Furthermore, the IG also experienced significantly superior improvement in UI symptoms compared with those who received only reality of care in Germany. A further confirmatory RCT is required to substantiate these oberservations.

## Supplementary Information

Below is the link to the electronic supplementary material.

Supplementary file1 (DOCX 567 KB)

Supplementary file2 (DOCX 34 KB)

## Data Availability

The data that support the main findings of this study are available in manuscript and supplementals. Additional data are available from the corresponding author upon request.
