# Supplementary material for: Digital Therapeutic FEMANEA® for Women with Stress and Mixed Urinary Incontinence: A Randomized Controlled Pilot Study
Source: Int Urogynecol J. 2026 Apr 30;37(9):2941–51. doi: 10.1007/s00192-026-06670-z (PMC13649793; doi:10.1007/s00192-026-06670-z)
Supplement: Supplementary file 1 [file 192_2026_6670_MOESM1_ESM.docx]

**Supplemental**

[Table 2 Inclusion and exclusion criteria](#_Toc196289854)

[Table 3 Chronic disease](#_Toc196289855)

[Table 4 Pre-treatment of urinary incontinence at T0](#_Toc196289856)

[Table 5 Concomitant treatments and medical assistance](#_Toc196289857)

[Table 6 Concomitant medication](#_Toc196289858)

[Table 7 Proportion of patients who were prescribed physiotherapy and T0 and received it at T1 and T2](#_Toc196289859)

[Table 8 ICIQ UI-SF and KHQ S2 at T0, T1 and T2](#_Toc196289860)

[Table 9 Frequency and amount of involuntary urinary loss at T0, T1 and T2](#_Toc196289861)

Table 2 Inclusion and exclusion criteria

| **Inclusion criteria** | **Exclusion criteria** |
| --- | --- |
| - Adult Women (≥ 18 years) - Pure SUI (ICD-10 code: N39.3) or MUI with predominant stress symptoms (ICD-10 code: N39.47, N39.48) - Symptoms corresponding to severity level 1 or 2 according to Ingelman-Sundberg - Ability of the participant to understand the nature and scope of the data analysis and the training program - Fulfillment of the technical requirements (Internet access with recommended browser: Edge, Chrome, Safari; playback medium: tablet, smartphone, PC, smart TV, laptop, etc.) - Availability of written consent for data analysis | - Pure urge incontinence (N39.42) or MUI with predominant urge symptoms, overflow incontinence (N39.41) and extraurethral urinary incontinence (N39.43) - Stress incontinence (N39.3) > severity level 2 and/or prolapse of the genital organs that is not treated with pessary therapy > grade 2 - Previous urinary incontinence surgery or surgical correction of urinary incontinence (within one year)/more than one surgical procedure for urinary incontinence - Hematuria or a history of more than three urinary tract infections in the past year - Congenital defects or abnormalities associated with incontinence - Existing bladder or rectal fistulas - Gynecological or urological tumor disease currently being treated with chemotherapy or radiotherapy - Interstitial cystitis (N30.1) or chronic pelvic pain - Current symptomatic pelvic floor prolapse - Patients scheduled to undergo surgery within 90 days of admission - Patients including the first six weeks after birth or in the postpartum period - Use of a comparable app at the same time or planned within the next 90 days or participation in a competing intervention study - Insufficient reading ability or lack of understanding of the German language - Mental or physical impairments that prevent correct understanding or execution of the exercises - Impaired vision. If the impairment is correctable (e.g., nearsightedness), participation is possible with appropriate aids (e.g., glasses) - Cardiovascular diseases where physical activity is contraindicated - Acute infection (e.g. cold, fever, or chills) - Pregnancy |

Table 3 Chronic disease

|  | All patients  n=74 | CG  n=40 | IG  n=34 |
| --- | --- | --- | --- |
| Patients with chronic diseases | 52 (70.3%) | 25 (62.5%) | 27 (79.4%) |
| Autoimmune diseases^1^ | 8 (10.8%) | 4 (10.0%) | 4 (11.8%) |
| Skin diseases and allergies | 9 (12.2%) | 5 (12.5%) | 4 (11.8%) |
| Cardiovascular diseases | 11 (14.9%) | 5 (12.5%) | 6 (17.6%) |
| Hormonal disorders^2^ | 21 (28.4%) | 11 (27.5%) | 10 (29.4%) |
| Cancer^3^ | 2 (2.7%) | 1 (2.5%) | 1 (2.9%) |
| Lung diseases | 4 (5.4%) | 2 (5.0%) | 2 (5.9%) |
| Gastrointestinal diseases | 1 (1.4%) | 1 (2.5%) | 0 (0.0%) |
| Neurological and mental disorders | 8 (10.8%) | 4 (10.0%) | 4 (11.8%) |
| Orthopaedic and musculosketal diseases | 7 (9.5%) | 3 (7.5%) | 4 (11.8%) |
| Metabolic disorders^4^ | 6 (8.1%) | 3 (7.5%) | 3 (8.8%) |
| Urological diseases^5^ | 4 (5.4%) | 3 (7.5%) | 1 (2.9%) |
| Other | 6 (8.1%) | 5 (12.5%) | 1 (2.9%) |

CG=control group, IG=intervention group ^1^ lupus erythematosus, Hashimoto's disease ^2^ hyperuricaemia, hypothyroidism, progesterone deficiency ^3^ breast cancer, skin cancer ^4^ obesity, dyslipidaemia, gestational diabetes, hyperinsulinism ^5^ chronic cystitis, pelvic organ prolapse, double kidneys.

Table 4 Pre-treatment of urinary incontinence at T0

|  | All patients  n=74 | CG  n=40 | IG  n=34 |
| --- | --- | --- | --- |
| Patients with pre-treatment | 20 (27.0%) | 9 (22.5%) | 11 (32.4%) |
| Patients without pre-treatment | 54 (73.0%) | 31 (77.5%) | 23 (67.6%) |
| Anticholinergics | 4 (20.0%) | 0 (0.0%) | 4 (36.4%) |
| Biofeedback device | 5 (25.0%) | 2 (22.2%) | 3 (27.3%) |
| Contam tempon/suportice tampons/vaginal tampons | 3 (15.0%) | 0 (0.0%) | 3 (27.3%) |
| Kegel training | 3 (15.0%) | 3 (33.3%) | 0 (0.0%) |
| Surgical therapy | 1 (5.0%) | 1 (11.1%) | 0 (0.0%) |
| Pessary/Uretrapessary | 3 (15.0%) | 1 (11.1%) | 2 (18.2%) |
| Physiotherapy and pelvic floor muscle training | 7 (35.0%) | 3 (33.3%) | 4 (36.4%) |
| SSNRI | 1 (5.0%) | 1 (11.1%) | 0 (0.0%) |
| Oestrogenisation | 3 (15.0%) | 2 (22.2%) | 1 (9.1%) |
| Other | 3 (15.0%) | 0 (0.0%) | 3 (27.3%) |

CG=control group, IG=intervention group, n=number, SSNRI=serotonin-norepinephrin reuptake inhibitor.

Table 5 Concomitant treatments and medical assistance

|  | All patients  n=74 | | CG  n=40 | IG  n=34 |
| --- | --- | --- | --- | --- |
| Patients with concomitant treatments and medical assistance | | | | |
| T0 | 59 (79.7%) | | 31 (77.5%) | 28 (82.4%) |
| T1 | 61 (82.4%) | | 32 (80.0%) | 29 (85.3%) |
| T2 | 63 (85.1%) | | 34 (85.0%) | 29 (85.3%) |
| Oestrogenisation | | | | |
| T0 | 5 (6.8%) | 2 (5.0%) | | 3 (8.8%) |
| T1 | 5 (6.8%) | 2 (5.0%) | | 3 (8.8%) |
| T2 | 5 (6.8%) | 2 (5.0%) | | 3 (8.8%) |
| Pessary | | | | |
| T0 | 5 (6.8%) | 2 (5.0%) | | 3 (8.8%) |
| T1 | 5 (6.8%) | 2 (5.0%) | | 3 (8.8%) |
| T2 | 5 (6.8%) | 2 (5.0%) | | 3 (8.8%) |
| Vaginal tampons | | | | |
| T0 | 6 (8.1%) | 2 (5.0%) | | 4 (11.8%) |
| T1 | 6 (8.1%) | 2 (5.0%) | | 4 (11.8%) |
| T2 | 6 (8.1%) | 2 (5.0%) | | 4 (11.8%) |
| Pelvic floor muscle training | | | | |
| T0 | 1 (1.4%) | 0 (0.0%) | | 1 (2.9%) |
| T1 | 2 (2.7%) | 0 (0.0%) | | 2 (5.9%) |
| T2 | 2 (2.7%) | 0 (0.0%) | | 2 (5.9%) |
| Biofeedback | | | | |
| T0 | 4 (5.4%) | 2 (5.0%) | | 2 (5.9%) |
| T1 | 5 (6.8%) | 2 (5.0%) | | 3 (8.8%) |
| T2 | 6 (8.1%) | 2 (5.0%) | | 4 (11.8%) |
| Electrical stimulation | | | | |
| T0 | 8 (10.8%) | 4 (10.0%) | | 4 (11.8%) |
| T1 | 8 (10.8%) | 4 (10.0%) | | 4 (11.8%) |
| T2 | 8 (10.8%) | 4 (10.0%) | | 4 (11.8%) |
| HIFEM energy | | | | |
| T0 | 2 (2.7%) | 2 (5.0%) | | 0 (0.0%) |
| T1 | 2 (2.7%) | 2 (5.0%) | | 0 (0.0%) |
| T2 | 2 (2.7%) | 2 (5.0%) | | 0 (0.0%) |
| Physiotherapy | | | | |
| T0 | 2 (2.7%) | 2 (5.0%) | | 0 (0.0%) |
| T1 | 2 (2.7%) | 2 (5.0%) | | 0 (0.0%) |
| T2 | 3 (4.1%) | 3 (7.5%) | | 0 (0.0%) |
| Digital assistance | | | | |
| T0 | 0 (0.0%) | 0 (0.0%) | | 0 (0.0%) |
| T1 | 0 (0.0%) | 0 (0.0%) | | 0 (0.0%) |
| T2 | 1 (1.4%) | 1 (2.5%) | | 0 (0.0%) |
| Other^[1]^ | | | | |
| T0 | 2 (2.7%) | 1 (2.5%) | | 1 (2.9%) |
| T1 | 2 (2.7%) | 1 (2.5%) | | 1 (2.9%) |
| T2 | 4 (5.4%) | 2 (5.0%) | | 2 (5.9%) |
| Incontinence pads/period lingerie | | | | |
| T0 | 53 (71.6%) | 28 (70.0%) | | 25 (73.5%) |
| T1 | 55 (74.3%) | 30 (75.0%) | | 25 (73.5%) |
| T2 | 58 (78.4%) | 32 (80.0%) | | 26 (76.5%) |

T0=1+7 days, T1=45±5 days, T2=95±5 days, CG=control group, IG=intervention group, n=number, HIFEM=High-Intensity Focused Electromagnetic, ^1^ Kentara plasters, YouTube videos, osteopathy, cones. Note: Missing data: for T1: n=1 (IG:1; CG:0), for T2: n=4 (IG:2; CG:2).

Table 6 Concomitant medication

|  | All patients  n=74 | CG  n=40 | IG  n=34 |
| --- | --- | --- | --- |
| Receive medication | | | |
| T0 | 42 (56.8%) | 19 (47.5%) | 23 (67.6%) |
| T1 | 43 (58.1%) | 19 (47.5%) | 24 (70.6%) |
| T2 | 44 (59.5%) | 20 (50.0%) | 24 (70.6%) |
| Analgesics | | | |
| T0 | 4 (5.4%) | 1 (2.5%) | 3 (8.8%) |
| T1 | 4 (5.4%) | 1 (2.5%) | 3 (8.8%) |
| T2 | 4 (5.4%) | 1 (2.5%) | 3 (8.8%) |
| Antiarrhythmics | | | |
| T0 | 2 (2.7%) | 1 (2.5%) | 1 (2.9%) |
| T1 | 2 (2.7%) | 1 (2.5%) | 1 (2.9%) |
| T2 | 2 (2.7%) | 1 (2.5%) | 1 (2.9%) |
| Anticolinergics | | | |
| T0 | 2 (2.7%) | 0 (0%) | 2 (5.9%) |
| T1 | 2 (2.7%) | 0 (0%) | 2 (5.9%) |
| T2 | 2 (2.7%) | 0 (0%) | 2 (5.9%) |
| Antidiabetics | | | |
| T0 | 1 (1.4%) | 0 (0.0%) | 1 (2.9%) |
| T1 | 1 (1.4%) | 0 (0.0%) | 1 (2.9%) |
| T2 | 1 (1.4%) | 0 (0.0%) | 1 (2.9%) |
| Antihistamines | | | |
| T0 | 3 (4.1%) | 1 (2.5%) | 2 (5.9%) |
| T1 | 3 (4.1%) | 1 (2.5%) | 2 (5.9%) |
| T2 | 3 (4.1%) | 1 (2.5%) | 2 (5.9%) |
| Antihypertensives | | | |
| T0 | 8 (10.8%) | 3 (7.5%) | 5 (14.7%) |
| T1 | 8 (10.8%) | 3 (7.5%) | 5 (14.7%) |
| T2 | 8 (10.8%) | 3 (7.5%) | 5 (14.7%) |
| Anti-infectives | | | |
| T0 | 1 (1.4%) | 1 (2.5%) | 0 (0.0%) |
| T1 | 3 (4.1%) | 2 (5.0%) | 1 (2.9%) |
| T2 | 3 (4.1%) | 2 (5.0%) | 1 (2.9%) |
| Respiratory medication | | | |
| T0 | 2 (2.7%) | 2 (5.0%) | 0 (0.0%) |
| T1 | 1 (1.4%) | 1 (2.5%) | 0 (0.0%) |
| T2 | 1 (1.4%) | 1 (2.5%) | 0 (0.0%) |
| Blood thinner | | | |
| T0 | 3 (4.1%) | 2 (5.0%) | 1 (2.9%) |
| T1 | 3 (4.1%) | 2 (5.0%) | 1 (2.9%) |
| T2 | 4 (5.4%) | 3 (7.5%) | 1 (2.9%) |
| Cholesterol reducer | | | |
| T0 | 3 (4.1%) | 1 (2.5%) | 2 (5.9%) |
| T1 | 3 (4.1%) | 1 (2.5%) | 2 (5.9%) |
| T2 | 4 (5.4%) | 2 (5.0%) | 2 (5.9%) |
| Immunmodulators | | | |
| T0 | 1 (1.4%) | 0 (0%) | 1 (2.9%) |
| T1 | 1 (1.4%) | 0 (0%) | 1 (2.9%) |
| T2 | 1 (1.4%) | 0 (0%) | 1 (2.9%) |
| Gastrointestinal^1^ | | | |
| T0 | 3 (4.1%) | 3 (7.5%) | 0 (0%) |
| T1 | 3 (4.1%) | 3 (7.5%) | 0 (0%) |
| T2 | 3 (4.1%) | 3 (7.5%) | 0 (0%) |
| Food supplements or vitamines and mineral supplements | | | |
| T0 | 5 (6.8%) | 4 (10.0%) | 1 (2.9%) |
| T1 | 5 (6.8%) | 4 (10.0%) | 1 (2.9%) |
| T2 | 5 (6.8%) | 4 (10.0%) | 1 (2.9%) |
| Psychotropic drugs | | | |
| T0 | 6 (8.1%) | 2 (5.0%) | 4 (11.8%) |
| T1 | 5 (6.8%) | 1 (2.5%) | 4 (11.8%) |
| T2 | 5 (6.8%) | 1 (2.5%) | 4 (11.8%) |
| Thyroid hormones | | | |
| T0 | 28 (37.8%) | 15 (37.5%) | 13 (38.2%) |
| T1 | 28 (37.8%) | 15 (37.5%) | 13 (38.2%) |
| T2 | 28 (37.8%) | 15 (37.5%) | 13 (38.2%) |
| Uricostatic drugs | | | |
| T0 | 2 (2.7%) | 1 (2.5%) | 1 (2.9%) |
| T1 | 2 (2.7%) | 1 (2.5%) | 1 (2.9%) |
| T2 | 2 (2.7%) | 1 (2.5%) | 1 (2.9%) |
| Female sex hormones | | | |
| T0 | 7 (9.5%) | 2 (5.0%) | 5 (14.7%) |
| T1 | 7 (9.5%) | 2 (5.0%) | 5 (14.7%) |
| T2 | 8 (10.8%) | 3 (7.5%) | 5 (14.7%) |
| Cytostatics | | | |
| T0 | 1 (1.4%) | 1 (2.5%) | 0 (0%) |
| T1 | 1 (1.4%) | 1 (2.5%) | 0 (0%) |
| T2 | 1 (1.4%) | 1 (2.5%) | 0 (0%) |
| Others | | | |
| T0 | 7 (9.5%) | 5 (12.5%) | 2 (5.9%) |
| T1 | 7 (9.5%) | 5 (12.5%) | 2 (5.9%) |
| T2 | 7 (9.5%) | 5 (12.5%) | 2 (5.9%) |

T0=1+7 days, T1=45±5 days, T2=95±5 days, CG=control group, IG=intervention group, n=number, ^1^ pan-toprazole and mesalazine. Note: Missing data: for T1: n=1 (IG:1; CG:0), for T2: n=4 (IG:2; CG:2).

Table 7 Proportion of patients who were prescribed physiotherapy and T0 and received it at T1 and T2

| Physiotherapy | All patients  n=74 | CG  n=40 | IG  n=34 | p-value *  CG vs IG |
| --- | --- | --- | --- | --- |
| T0 (Prescription) | 52 (70.3%) | 29 (72.5%) | 23 (67.6%) | 0.841 |
| T1 (Physiotherapy received) | 20 (27.0%) | 13 (32.5%) | 7 (20.6%) | 0.416 |
| T2 (Physiotherapy received) | 29 (39.2%) | 19 (47.5%) | 10 (29.4%) | 0.179 |

T0=1+7 days, T1=45±5 days, T2=95±5 days, CG=control group, IG=intervention group, n=number, *Calculated using the chi-square test. Note: Missing data: for T1: n=1 (IG:1; KG:0), for T2: n=4 (IG:2; KG:2).

Table 8 ICIQ UI-SF and KHQ S2 at T0, T1 and T2

| ICIQ-UI SF at observation time point [M (SD) n] | | | | | |
| --- | --- | --- | --- | --- | --- |
|  | All patients | CG | IG | Cohen’s d  CG vs IG | p-value*  CG vs IG |
| T0 | 9.89 (3.70) 74 | 9.20 (3.00) 40 | 10.71 (4.29) 34 | 0.407 | 0.091 |
| T1 | 8.66 (3.54) 73 | 8.38 (2.75) 40 | 9.00 (4.32) 33 | 0.172 | 0.475 |
| T2 | 8.27 (3.70) 70 | 8.32 (2.73) 38 | 8.22 (4.65) 32 | -0.025 | 0.918 |
| Δ (T0, T2)** | -1.47 (2.74) 70 | -0.84 (2.57) 38 | -2.22 (2.79) 32 | -0.513 | 0.037 |
| KHQ at observation time point [M (SD) n] | | | | | |
|  | All patients | CG | IG | Cohen’s d  CG vs IG | p-value*  CG vs IG |
| T0 | 50.45 (26.02)  74 | 45.00 (22.07)  40 | 56.86 (29.05)  34 | 0.460 | 0.056 |
| T1 | 46.58 (24.68)  73 | 43.33 (21.62)  40 | 50.51 (27.79)  33 | 0.288 | 0.231 |
| T2 | 38.10 (24.92)  70 | 40.35 (23.45)  38 | 35.42 (26.69)  32 | -0.196 | 0.419 |
| Δ (T0, T2)** | -11.90 (20.49)  70 | -5.26 (19.80)  38 | -19.79 (18.66)  32 | -0.755 | 0.002 |

T0=1+7 days, T1=45±5 days, T2=95±5 days, ICIQ-UI SF= International Consultation on Incontinence Questionnaire-Urinary Incontinence Short Form, KHQ= King's Health Questionnaire, IG=intervention group, CG=control group, M=mean, SD=standard deviation, n=number. *Calculated using t-test. **Calculated by first using Δ (T0, T2) per patient as a new variable and then taking the mean value from this. Statistically significant (p<0.05) intra-individual improvements from T0 to T2 are highlighted at T2, high scores of ICIG-UI SF or KHQ indicate a high incontinence burden.

Table 9 Frequency and amount of involuntary urinary loss at T0, T1 and T2

| Individual frequency of involuntary urinary loss (events per day) | | | | |
| --- | --- | --- | --- | --- |
|  | All patients | CG | IG | p-value*  CG vs IG |
|  | M (SD)  n | M (SD)  n | M (SD)  n |  |
| T0 | 0.66 (0.96)  74 | 0.60 (0.77)  40 | 0.74 (1.16)  34 | 0.564 |
| T1 | 0.45 (0.82)  69 | 0.50 (0.72)  37 | 0.40 (0.93)  32 | 0.626 |
| T2 | 0.23 (0.51)  70 | 0.31 (0.60)  38 | 0.15 (0.35)  32 | 0.168 |
| Δ (T0, T2)** | -0.35 (0.61)  70 | -0.32 (0.64)  38 | -0.39 (0.58)  32 | 0.677 |
| Individual amount of involuntary urinary loss [g / 24h] | | | | |
|  | All patients | CG | IG | p-value*  CG vs IG |
|  | M (SD)  n | M (SD)  n | M (SD)  n |  |
| T0 | 15.45 (42.89)  74 | 8.92 (17.86)  40 | 23.14 (59.82)  34 | 0.189 |
| T1 | 13.44 (34.02)  70 | 10.25 (27.49)  38 | 17.23 (40.57)  32 | 0.412 |
| T2 | 13.73 (40.83)  70 | 14.39 (46.8)  38 | 12.95 (33.09)  32 | 0.881 |
| Δ (T0, T2)** | -1.1 (32.51)  70 | 5.16 (30.49)  38 | -8.55 (33.72)  32 | 0.082 |

T0=1+7 days, T1=45±5 days, T2=95±5 days, IG=intervention group, CG=control group, M=mean, SD=standard deviation, n=number *Calculated using t-test. **Calculated by first using Δ (T0, T2) per patient as a new variable and then taking the mean value from this. Frequency: statistically significant (p<0.05) intraindividual improvements from T0 to T2 are highlighted at T2, amount: no statistically significant intraindividual improvement from T0 to T2.

Table 10 Mixed Linear Model Regression Results of ICIQ-UI SF

**Table 11** Mixed Linear Model Regression Results of KHQ S2

**Table 12** Mixed Linear Model Regression Results of amount of involuntary urinary loss
